# Transsphenoidal surgery for pituitary adenoma: indications and outcomes

**DOI:** 10.3325/cmj.2012.53.639

**Published:** 2012-12

**Authors:** Tina Dušek, Ante Melada, Josip Paladino, Darko Kaštelan

**Affiliations:** 1 University of Zagreb School of Medicine, Zagreb, Croatia; 2 Department of Internal Medicine, Division of Endocrinology, University Hospital Center Zagreb, Zagreb, Croatia; 3 Department of Neurosurgery, University Hospital Center Zagreb, Zagreb, Croatia

To the Editor: We read with great interest the article by Marić et al (1) on the outcomes of pure endoscopic transsphenoidal surgery (PEETS). Since PEETS is a relatively new surgical technique, data on its effectiveness and safety are very valuable, which makes this article an important contribution to the field. However, we have some concerns about the protocol of the study regarding the biochemical criteria for remission of acromegaly, assessment of pituitary function, and the indications for surgical treatment of prolactinoma.

In order to estimate the acromegaly remission, the authors performed the measurement of insulin-like growth factor-1 (IGF-1) on the seventh postoperative day. In our opinion, this might be too early because IGF-1 may remain elevated for months after a successful surgery. According to the Consensus on Criteria for Cure of Acromegaly (2,3), successfulness of surgical treatment of acromegaly is defined by the adequate suppression of the growth hormone after the glucose load and normalization of IGF-1 three to six months after surgery. The level of IGF-1 in the immediate postoperative period might therefore be misleading in the estimation of acromegaly remission.

With regard to the assessment of pituitary function, the usual criterion for the postoperative improvement of hypogonadism in women of reproductive age is the resumption of the menstrual cycle, rather than the normalization of the estrogen level on the seventh postoperative day, as used in the study. Moreover, in the assessment of the adrenal function, the authors measured urinary free cortisol in some patients who were taking hydrocortisone replacement therapy at the same time. We agree with the authors that the insulin tolerance test (ITT), which is the gold standard in the assessment of the hypothalamo-pituitary-adrenal (HPA) axis, is a rather demanding procedure. Therefore in practice, the simpler, short synacthen test is usually applied as an alternative to ITT. To the best of our knowledge, the measurement of urinary free cortisol in patients under hydrocortisone therapy does not have proven specificity and sensitivity for the detection of HPA axis abnormalities.

Furthermore, the number of patients in the study who underwent pituitary surgery for prolactinoma was surprisingly high. Surgical management of prolactinoma has its controversies: potential surgical and endocrinological complications, as well as the recurrence of hyperprolactinemia. On the other hand, dopamine agonists are remarkably effective in normalization of serum prolactin level, restoration of gonadal function, and reduction of tumor size in patients with prolactinoma (4). We would like to emphasize that, according to the Endocrine Society Clinical Practice Guidelines (5) and our own experience, only a minority of patients with prolactinoma requires surgical treatment. In our cohort, for example, only one out of 62 patients treated for prolactinoma in the last three years required surgery. It is known that up to 40% of patients who have undergone an initial surgical remission might have a recurrence of the disease (6). Therefore, the authors’ conclusion that surgery leads to remission of microprolactinoma in 100% of cases should be taken with some caution as they did not present the data from the follow-up period.

In conclusion, because of insufficient research on PEETS, studies like this are highly valuable. Nevertheless, we appeal to the authors to use contemporary diagnostic tests and cutoff values in the evaluation of pituitary disorders in order to have the results comparable to those of other authors in the field.

## References

[1] Marić A, Kruljac I, Čerina V, Pećina HI, Šulentić P, Vrkljan M (2012). Endocrinological outcomes of pure endoscopic transsphenoidal surgery: a Croatian Referral Pituitary Center experience.. Croat Med J.

[2] Melmed S, Colao A, Barkan A, Molitch M, Grossman AB, Kleinberg D (2009). Guidelines for acromegaly management: an update.. Clin Endocrinol Metab.

[3] Giustina A, Chanson P, Bronstein MD, Klibanski A, Lamberts S, Casanueva FF (2010). A Consensus on Criteria for Cure of Acromegaly.. J Clin Endocrinol Metab.

[4] Casanueva FF, Molitch ME, Schlechte JA, Abs R, Bonert V, Bronstein MD (2006). Guidelines of the Pituitary Society for the diagnosis and management of prolactinomas.. Clin Endocrinol (Oxf).

[5] Melmed S, Casanueva FF, Hoffman AR, Kleinberg DL, Montori VM, Schlechte JA (2011). Diagnosis and Treatment of Hyperprolactinemia: An Endocrine Society Clinical Practice Guideline. J Clin Endocrinol Metab.

[6] Ciccarelli E, Ghigo E, Miola C, Gandini G, Muller EE, Camanni F (1990). Long-term follow-up of ‘cured’ prolactinoma patients after successful adenomectomy.. Clin Endocrinol (Oxf).

